# Safety and efficacy of co-careldopa as an add-on therapy to occupational and physical therapy in patients after stroke (DARS): a randomised, double-blind, placebo-controlled trial

**DOI:** 10.1016/S1474-4422(19)30147-4

**Published:** 2019-06

**Authors:** Gary A Ford, Bipin B Bhakta, Alastair Cozens, Suzanne Hartley, Ivana Holloway, David Meads, John Pearn, Sharon Ruddock, Catherine M Sackley, Eirini-Christina Saloniki, Gillian Santorelli, Marion F Walker, Amanda J Farrin

**Affiliations:** a Oxford University Hospitals NHS Foundation Trust, University of Oxford, Oxford, UK; b The Academic Department of Rehabilitation Medicine, Leeds Institute of Rheumatic and Musculoskeletal Medicine, University of Leeds, Leeds, UK; c Clinical Trials Research Unit, Leeds Institute of Clinical Trials Research, University of Leeds, Leeds, UK; d Academic Unit of Health Economics, Leeds Institute of Health Sciences, University of Leeds, Leeds, UK; e NHS Grampian, Aberdeen, UK; f School of Population and Environmental Sciences, Faculty of Life Science and Medicine, King's College London, London, UK; g Centre for Health Services Studies and Personal Social Services Research Unit, University of Kent, Canterbury, UK; h Rehabilitation and Ageing, Queens Medical Centre, University of Nottingham, Nottingham, UK

## Abstract

**Background:**

Dopamine is a key modulator of striatal function and learning and might improve motor recovery after stroke. Previous small trials of dopamine agonists after stroke provide equivocal evidence of effectiveness on improving motor recovery. We aimed to assess the safety and efficacy of co-careldopa plus routine occupational and physical therapy during early rehabilitation after stroke.

**Methods:**

This double-blind, multicentre, randomised controlled trial of co-careldopa versus placebo in addition to routine NHS occupational and physical therapy was done at 51 UK NHS acute inpatient stroke rehabilitation services. We recruited patients with new or recurrent clinically diagnosed ischaemic or haemorrhagic (excluding subarachnoid haemorrhage) stroke 5–42 days before randomisation, who were unable to walk 10 m or more, had a score of less than 7 points on the Rivermead Mobility Index, were expected to need rehabilitation, and were able to access rehabilitation after discharge from hospital. Participants were assigned (1:1) using stratified random blocks to receive 6 weeks of oral co-careldopa or matched placebo in addition to routine NHS physiotherapy and occupational therapy. The initial two doses of co-careldopa were 62·5 mg (50 mg of levodopa and 12·5 mg of carbidopa) and the remaining doses were 125 mg (100 mg of levodopa and 25 mg of carbidopa). Participants were required to take a single oral tablet 45–60 min before physiotherapy or occupational therapy session. The primary outcome was ability to walk independently, defined as a Rivermead Mobility Index score of 7 or more, at 8 weeks. Primary and safety analyses were done in the intention-to-treat population. The trial is registered on the ISRCTN registry, number ISRCTN99643613.

**Findings:**

Between May 30, 2011, and March 28, 2014, of 1574 patients found eligible, 593 (mean age 68·5 years) were randomly assigned to either the co-careldopa group (n=308) or to the placebo group (n=285), on an average 18 days after stroke onset. Primary outcome data were available for all 593 patients. We found no evidence that the ability to walk independently improved with co-careldopa (125 [41%] of 308 patients) compared with placebo (127 [45%] of 285 patients; odds ratio 0·78 [95% CI 0·53–1·15]) at 8 weeks. Mortality at 12 months did not differ between the two groups (22 [7%] vs 17 [6%]). Serious adverse events were largely similar between groups. Vomiting during therapy sessions, after taking the study drug, was the most frequent adverse event and was more frequent in the co-careldopa group than the placebo group (19 [6·2%] *vs* 9 [3·2%]).

**Interpretation:**

Co-careldopa in addition to routine occupational and physical therapy does not seem to improve walking after stroke. Further research might identify subgroups of patients with stroke who could benefit from dopaminergic therapy at different doses or times after stroke with more intensive motor therapy.

**Funding:**

Medical Research Council.

## Introduction

Studies of the brain structures involved in learning suggest that the basal ganglia and dopamine play a key part in the acquisition of motor skills. Dopamine is a key modulator of striatal function and might contribute to motor recovery after stroke.^1^, ^2^ Preclinical studies^3^, ^4^ suggest that the potential mechanisms of action of dopamine in improving motor learning are through potentiating drive and arousal in conditioned learning and up-regulation of glutaminergic transmission, which modulates synaptic efficacy. Levodopa is an orally-administered dopamine precursor that crosses the blood–brain barrier before being metabolised to dopamine. Co-careldopa is an established treatment for Parkinson's disease that combines levodopa with carbidopa, a peripheral DOPA-decarboxylase inhibitor that maximises the central bioavailability of levodopa.

One systematic review^5^ of clinical trials investigated the use of dopamine agonists to enhance motor recovery from stroke and concluded that the evidence was insufficient. Seven small trials of dopamine agonists after stroke have provided equivocal evidence on motor recovery.^6^, ^7^, ^8^, ^9^, ^10^, ^11^, ^12^ Trials were of variable quality, with small sample sizes,^8^, ^10^ short follow-up,^10^ single doses of co-careldopa,^10^ and recruitment of patients months or years after stroke.^8^, ^10^ Four of the seven trials showed improvement in a motor outcome.^6^, ^8^, ^9^, ^12^ Therefore, a larger, randomised controlled trial is needed to investigate whether levodopa enhances recovery from stroke.

**Research in context**

**Evidence before this study**

We did a systematic search of MEDLINE (1946–Sept 25, 2015), Embase (1996–Week 42, 2014), Embase Classic (1947–Sept 25, 2015), PsychINFO (1806–Sept 25, 2015), and the Cochrane Database of Systematic Reviews for randomised controlled trials and systematic reviews assessing dopaminergic therapy on motor recovery after stroke. The search included expanded terms relating to “stroke”, “dopamine”, and “rehabilitation” (appendix). Only one systematic review had examined the use of dopamine agonists to enhance motor recovery from stroke in humans. Two studies concerning the use of levodopa met the review inclusion criteria, neither of which showed evidence of a positive treatment effect with this drug. Seven other trials, not cited by the systematic review, addressed this question. These trials were of variable quality and reported mixed results. Many were limited by small sample sizes (n=10–100) or comparatively short follow-ups (15–180 days), or only single doses of co-careldopa were administered. Some recruited patients months or years after stroke. Several trials showed benefits of dopamine on motor outcomes; however, others found no improvement. These disparate findings have not thus far addressed the question whether pharmacological manipulation of the dopaminergic systems could be used to enhance the reacquisition of motor skills after stroke.

**Added value of the study**

We found no evidence that combining dopaminergic therapy with routine NHS occupational and physical therapy was effective in improving walking after stroke. Our study was limited by the 10% of patients who were lost to follow-up at 8 weeks; less than 10% of patients met the strict definition to be included in per-protocol analysis of the primary outcome; and the intensity of therapy delivered in DARS could have been insufficient. Despite these limitations, the findings seem to be robust and generalisable to patients with limited mobility in the first few weeks after stroke.

**Implications of all the available evidence**

The findings of our study appear consistent with smaller studies of dopaminergic therapy. A formal meta-analysis of trials of dopaminergic therapy in stroke recovery has not been done and given the wide range of different outcome measures in populations of patients with stroke recruited at different times after stroke, it might not be justified. Further research is needed to develop imaging and clinical markers that will allow identification of promising drug therapies that might enhance motor therapy for improving walking ability and arm function after stroke. Subgroups of patients with stroke who might benefit from dopaminergic therapy at different doses or times after stroke with more intensive motor therapy need to be identified.

To optimise efficacy of dopaminergic therapy and minimise adverse effects, administering levodopa before motor therapy to enhance brain dopamine concentrations during therapy is a logical strategy. We hypothesised that elevating central dopaminergic activity during motor therapy early after stroke onset would improve motor recovery, when the potential for recovery might be greater.

The DARS trial aims to assess whether giving a combination of co-careldopa for up to 6 weeks with routine occupational and physical therapy during early rehabilitation after stroke enhances the effect of conventional rehabilitation on the ability to walk independently for 10 m or more.^13^

## Methods

### Study design and participants

DARS was a multicentre, prospective, double-blind, randomised placebo-controlled trial of NHS physical therapy and occupational therapy treatment alone versus NHS physical therapy and occupational therapy with up to 6 weeks co-careldopa treatment for people with new or recurrent stroke admitted to acute stroke services in hospital. Recruitment was from 51 UK NHS acute inpatient stroke rehabilitation services with a community stroke rehabilitation service.

Eligible participants had new or recurrent clinically diagnosed ischaemic or haemorrhagic stroke 5–42 days before randomisation, could not walk independently for 10 m or more indoors, had a Rivermead Mobility Index (RMI; a 15-item scale that assesses functional mobility in gait, balance, and transfers after stroke)^14^ score of less than 7 (marked by a health-care professional), and were able to give written informed consent, access rehabilitation treatment within 5 days of hospital discharge, and were expected to be in hospital for administration of at least their first two trial medication doses. Exclusion criteria included diagnosis of Parkinson's disease or symptomatic orthostatic hypotension (panel). Detailed trial methods are described elsewhere^13^ and can be found in the trial protocol (appendix). Ethical approval was obtained through the UK National Research Ethics Service (10/H1005/6).

Panel **Inclusion and exclusion criteria**

**Inclusion criteria:**

• New or recurrent clinically diagnosed ischaemic or haemorrhagic (excluding subarachnoid haemorrhage) stroke within 5–42 days before randomisation
• Cannot walk 10 m or more indoors independently (ie, without use of physical assistance)
• Achieved a score of less than 7 points on the Rivermead Mobility Index, scored by a professional
• Expected to need rehabilitation treatment
• Aged 18 years and older
• Able to give informed consent
• Able to access continuity of rehabilitation treatment after discharge from hospital (ie, continuity of rehabilitation available within 5 days after discharge)
• Expected to be able to comply with the treatment schedule
• Expected to be in hospital for at least their first two doses of trial medication

**Exclusion criteria:**

• Not expected to survive for 2 months after stroke
• A diagnosis of Parkinson's disease, severe medical or surgical illness, or severe psychosis
• Known hypersensitivity or contraindications to co-careldopa
• Symptomatic orthostatic hypotension
• Required physical assistance from at least one person to walk before stroke due to pre-existing comorbidities (eg, heart failure or osteoarthritis)
• Pregnancy, lactation, or, in the case of women of child-bearing potential, unwillingness to use medically-approved contraception during treatment and for 1 month after treatment had finished
• Participation in another interventional drug or treatment therapy trial
• Inability to walk 10 metres or further indoors before stroke (with a walking aid if necessary, but without physical assistance, which, in this context, means help from one or more people)

### Randomisation and masking

Participants were randomly assigned (1:1) via a computer-generated programme, with the use of random permutated blocks of size four, to receive either co-careldopa or placebo. Stratification factors were centre, type of stroke (primary intracranial haemorrhage or infarct), and RMI score (0–3 or 4–7). Patients, clinicians, researchers, and trial staff were masked to treatment allocation, which was continued throughout the trial period and follow up. Potential participants were identified by National Institute for Health Research Stroke Research Network (SRN) staff in liaison with ward nurses and therapists.

### Procedures

Participants received either co-careldopa (Sinemet) or matching placebo before routine therapy sessions, for a maximum of 6 weeks. The content and number of sessions was variable according to patient need and was decided as part of the routine management of the patient. The initial two co-careldopa doses were both 62·5 mg (levodopa 50 mg; carbidopa 12·5 mg) and subsequent doses were 125 mg (levodopa 100 mg; carbidopa 25 mg). Participants took the study drug orally 45–60 min before physical therapy or occupational therapy sessions directed at motor skills (walking, transfers, dressing). Therapy staff recorded details of every therapy session delivered from day of randomisation to the last administration of co-careldopa or placebo or 6 weeks post-randomisation (whichever was sooner). This included timing of drug administration in relation to therapy, duration of therapy session (overall and by activity), and role of therapist present. Patients might not have been able to take the drug 45–60 min before the start of a therapy session. In these situations, the drug was administered immediately before the start of a session.

Baseline data were collected by the clinical research team from clinical records and via face-to-face administration of the questionnaires. All baseline data were collected before randomisation. Follow-up data and safety data were collected at 8 weeks, 6 months, and 12 months, face-to-face, in the participant's home, at the hospital, or at a community facility. Data were recorded on paper case report forms provided by the Clinical Trials Research Unit. Completion of the primary outcome measure (RMI) was via telephone when it was not possible to arrange a face-to-face visit.

### Outcomes

The primary outcome was the ability to walk at least 10 m independently at 8 weeks after randomisation, as measured by an RMI score or 7 or more and positive response to item 7 (walk at least 10 m independently with an aid if necessary, but no standby help).

Secondary outcomes were independent walking at 6 months and 12 months (RMI score ≥7), physical functioning (Nottingham Extended Activities Daily Living [NEADL],^15^, Barthel Index,^16^ ABILHAND Manual Ability Measure,^17^ modified Rankin Scale [mRS]),^18^ cost-effectiveness, pain (musculoskeletal symptoms and signs and pain [MSK-SSP] Manikin), cognition (Montreal Cognitive Assessment),^19^ mood (General Health Questionnaire 12 [GHQ-12]),^20^ fatigue (Fatigue Assessment Scale),^21^ and carer burden (Caregiver Burden Scale [CBS])^22^ at 8 weeks, 6 months, and 12 months.

Follow-up and safety data were collected face-to-face, where possible, by an independent, masked researcher. RMI assessment was completed via telephone if a face-to-face visit was not possible. Adverse events were collected until the 8-week follow up appointment. All serious adverse events were collected up to 30 days after the last dose of protocol treatment.

### Statistical analysis

Based on a previous study,^12^ 572 participants would provide 90% power at 5% significance to detect a 50% relative difference (13% absolute difference) between placebo and active treatment groups in the primary outcome, assuming that 26% of people in the placebo group achieve independent walking at 8 weeks, allowing for 5% mortality rate. Since trial monitoring indicated that the combined mortality rate and loss to follow-up was likely to exceed 10%, the sample size was increased to 590–600 participants.

We based analyses on the intention-to-treat population (ITT), with significance assessed at the 5% level. We included all randomly assigned participants in the primary ITT analysis, assuming those participants who died or were lost to follow-up were unable to walk independently. We used a multilevel logistic regression model, adjusting for stratification variables: age, sex, type of stroke, RMI baseline score, centre (fitted as a random effect), baseline prestroke NEADL and Barthel Index scores, and number of days from stroke to randomisation. We report parameter estimates or odds ratios (ORs), with 95% CIs and p values (fixed effects) or SEs (random effects). Sensitivity analyses assessed the robustness of the primary analysis. We included all randomly assigned participants in the safety analyses.

We analysed independent walking ability at 6 months and 12 months after randomisation in a similar way. We used stepwise multilevel linear regression to analyse secondary endpoints, except for mRS, for which we used a stepwise multilevel proportional odds logistic regression model. We used further analyses to assess the sensitivity of the conclusions of this analysis to non-compliance, using a staged definition based on whether and when the drug was taken, the amount of motor therapy, and the number of sessions.

We estimated the incremental cost per quality-adjusted life-year (QALY) from the UK health and social care perspective over 12 months using the within-trial economic evaluation. We based costs on a resource use questionnaire and utility derived from the European Quality of Life–Five Dimensions Questionnaire (EQ-5D) measure. We used multiple imputation to handle missing data. Non-parametric bootstrapping characterised uncertainty in the incremental cost-effectiveness ratio. All analyses were completed in SAS software, version 9.2.

### Role of funding source

The funder of the study had no role in the study design, data collection, data analysis or data interpretation, or writing of the report. The corresponding author had access to all the data in the study and had final responsibility for the decision to submit for publication.

## Results

During recruitment from May 30, 2011, to March 28, 2014, of 1547 eligible individuals, 593 (38%) were randomly assigned: 308 to the co-careldopa group and 285 to the placebo group (figure). Baseline demographic characteristics were balanced between the two groups (table 1).

**Figure:**
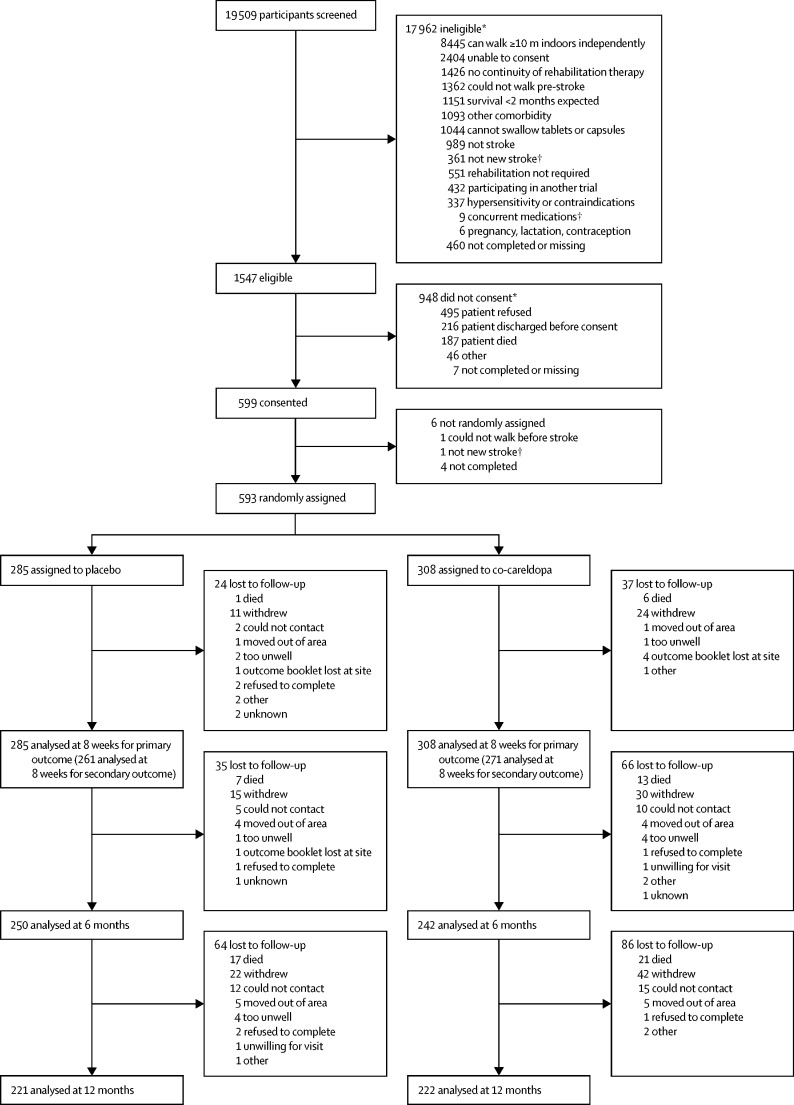
Trial profile *Categories are not mutually exclusive —ie, some patients had more than one exclusion criteria. †Initial screening categories.

**Table 1:** Baseline demographic and clinical characteristics

			**Co-careldopa (n=308)**	**Placebo (n=285)**
Age, years			67·5 (13·6)	69·6 (12·7)
Sex				
	Men		187 (61%)	177 (62%)
	Women		121 (39%)	108 (38%)
Ethnicity				
	White		289 (94%)	270 (95%)
	Asian		9 (3%)	8 (2%)
	Black		3 (1%)	5 (2%)
	Chinese		2 (1%)	1 (<1%)
	Mixed		2 (1%)	1 (<1%)
	Other		3 (1%)	0
Type of stroke				
	Infarction*		270 (88%)	238 (84%)
		Total Anterior Circulation	98 (36%)	63 (27%)
		Lacunar	58 (22%)	58 (24%)
		Partial Anterior Circulation	87 (32%)	91 (38%)
		Posterior Circulation	27 (10%)	25 (11%)
		Missing	..	1 (<1%)
	Primary haemorrhage		38 (12%)	47 (17%)
Thrombolysis received			62 (23%)	59 (25%)

Data are mean (SD) or n (%).

* Oxfordshire Community Stroke Project classification.

In total, 532 (90%) participants completed follow-up assessments at 8 weeks, 492 (83%) at 6 months, and 443 (75%) at 12 months (figure). Baseline characteristics of non-responders did not differ between groups at any timepoint, except at 6 months, when non-responders had higher prestroke NEADL scores in the co-careldopa group than in the placebo group (59·6 *vs* 52·5 points). During the trial, 14 551 motor therapy sessions were delivered (mean of 23·2 sessions [SD 14·4] per patient, and mean duration of 42·8 [15·1] min in the co-careldopa group; mean of 24·8 [12·5] sessions per patient, and mean duration of 43·1 [16·0] min in the placebo group). The average time spent on motor activities was 40 min [SD 15]. More participants in the placebo group than in the co-careldopa group received sufficient motor therapy (at least five therapy sessions and ≥20 min motor therapy in at least 80% therapy sessions; 257 [90%] of 285 patients *vs* 259 [84%] of 308 patients).

The study drug was taken per protocol (45–60 min before therapy) in 8006 (55%) of 14 451 therapy sessions and either less than 45 min or more than 60 min before therapy in a further 3843 (26%) sessions (appendix). Participants in the co-careldopa group received a mean of 20·7 study drug doses (SD 13·1), and the placebo group 22·4 (11·1). 14 (2%) participants (ten in the co-careldopa group and four in the placebo group) did not receive any study drug doses or therapy, mainly due to withdrawal before therapy.

The primary analysis did not provide evidence of a difference in the proportion of participants walking independently at 8 weeks (125 [41%] of 308 patients in the co-careldopa group *vs* 127 [45%] of 285 patients in the placebo group; OR 0·78 [95% CI 0·53–1·15]; table 2). Sensitivity analyses supported the conclusions from the primary analysis (appendix). A per-protocol analysis could not be done because of the small number of patients remaining in the per-protocol population. Complier-average causal effect analyses, which used a staged definition of intervention compliance, supported the conclusions of the primary analysis (appendix).

**Table 2:** Assessments at baseline, 8 weeks, 6 months, and 12 months after randomisation

		**Baseline**		**8 weeks**		**6 months**		**12 months**	
		Co-careldopa (n=308)	Placebo (n=285)	Co-careldopa (n=271)	Placebo (n=261)	Co-careldopa (n=242)	Placebo (n=250)	Co-careldopa (n=222)	Placebo (n=221)
Able to walk independently		10 (3%)	7 (3%)	125/308 (41%)	127/285 (45%)	159/308 (52%)	152/285 (53%)	159/308 (52%)	162/285 (57%)
	Odds ratio (95% CI); p value	..	..	..	0·78 (0·53–1·15); 0·212	..	..	..	..
Patient-reported RMI (as continuous)		2·4 (2·2)	2·5 (2·2)	6·8 (4·2)	7·0 (4·2)	8·3 (4·6)	8·1 (4·5)	8·7 (4·7)	8·5 (4·6)
	Adjusted mean difference (95% CI); p value	..	..	..	−0·35 (−0·89 to 0·19); 0·198	..	0·14 (−0·50 to 0·79); 0·662	..	0·17 (−0·54 to 0·88); 0·637
NEADL*		59·0 (11·0)	58·6 (12·4)	21·0 (17·7)	20·0 (15·8)	27·2 (18·2)	27·3 (18·1)	30·4 (19·4)	29·8 (18·9)
	Adjusted mean difference (95% CI); p value	..	..	..	1·02 (−1·27 to 3·30); 0·382	..	0·027 (−2·72 to 2·78); 0·985	..	1·04 (−1·56, 3·64); 0·434
Barthel Index		7·7 (3·8)	7·8 (3·7)	12·9 (5·1)	13·2 (4·9)	14·0 (5·1)	14·4 (5·1)	14·4 (5·4)	14·6 (5·1)
	Adjusted mean difference (95% CI); p value	..	..	..	−0·22 (−0·87 to 0·43); 0·511	..	−0·33 (−1·08 to 0·41); 0·378	..	−0·22 (−1·04 to 0·59); 0·591
ABILHAND, logits		0·8 (3·9)	0·3 (1·8)	0·2 (2·3)	0·4 (2·2)	0·1 (2·4)	0·3 (2·5)	0·2 (2·6)	0·4 (2·6)
	Adjusted mean difference (95% CI); p value	..	..	..	−0·10 (−0·46 to 0·26); 0·585	..	−0·15 (−0·57 to 0·27); 0·478	..	−0·16 (−0·59 to 0·28); 0·479
GHQ-12		19·4 (6·7)	19·3 (7·0)	16·9 (7·2)	16·4 (6·6)	15·1 (7·0)	16·3 (6·8)	14·0 (6·8)	14·4 (7·2)
	Adjusted mean difference (95% CI); p value	..	..	..	0·24 (−0·88 to 1·36); 0·677	..	−1·33 (−2·57 to 0·10); 0·035	..	−0·77 (−2·01 to 0·52); 0·241
	No sign of psychological distress	91 (30%)	94 (33%)	128 (42%)	121 (43%)	139 (45%)	125 (44%)	152 (49%)	133 (47%)
FAS		NA	NA	25·1 (7·6)	24·8 (7·4)	25·9 (8·1)	25·4 (7·6)	24·9 (8·3)	24·5 (8·2)
mRS†		NA	NA	..	..	..	..	NA	NA
	0	..	..	3 (1·0)	1 (0·4)	1 (0·3)	2 (0·7)	..	..
	1	..	..	15 (4·9)	11 (3·9)	29 (9·4)	25 (8·8)	..	..
	2	..	..	24 (7·8)	30 (10·5)	23 (7·5)	30 (10·5)	..	..
	3	..	..	101 (32·8)	114 (40·0)	123 (39·9)	128 (44·9)	..	..
	4	..	..	95 (30·8)	79 (27·7)	41 (13·3)	47 (16·5)	..	..
	5	..	..	34 (11·0)	34 (11·9)	27 (8·8)	16 (5·6)	..	..
	6	..	..	6 (1·9)	1 (0·4)	6 (1·9)	4 (1·4)	..	..
	Odds ratio (95% CI); p value	..	..	..	0·87 (0·63 to 1·21); 0·404	..	0·81 (0·57 to 1·14); 0·226	..	Not reported
MoCA		20·0 (6·6)	20·5 (6·0)	22·4 (6·3)	22·9 (5·5)	23·1 (6·2)	23·6 (5·5)	23·1 (5·9)	23·5 (5·6)
	Adjusted mean difference (95% CI); p value	..	..	..	−0·16 (−0·75 to 0·43); 0·592	..	−0·27 (−0·96 to 0·42); 0·445	..	−0·19 (−0·95 to 0·56); 0·613
Caregiver Burden Scale†		NA	NA	43·0 (13·4)	46·6 (13·9)	44·6 (13·6)	49·1 (14·7)	44·6 (15·1)	51·8 (15·3)
	Adjusted mean difference (95% CI); p value	..	..	..	−4·55 (0·14 to 8·96); 0·043	..	−4·99 (0·173 to 9·811); 0·042	..	−7·17 (1·70 to 12·64); 0·011
Number of carer respondents		..	..	74	72	62	65	50	107

Data are mean (SD) or n (%), unless otherwise specified. The proportion of patients included for the secondary analyses are based on number of patients randomly assigned. RMI=Rivermead Mobility Index; a higher score indicates increasing ability to walk independently. NEADL=Nottingham Extended Activities of Daily Living Scale; a higher score indicates greater independence. ABILHAND=a Manual Ability Measure; raw scores are converted into a linear measure and expressed as logits; a higher number logit indicates greater patient's perceived ability. GHQ-12=General Health Questionnaire 12; a higher score indicates worse health. FAS=Fatigue Assessment Scale; a higher score indicates more severe fatigue. mRS=modified Rankin Scale; a higher score indicates greater levels of current functional independence; patients who die are given a score of 6. MoCA=Montreal Cognitive Assessment; score <26 indicates cognitive impairment. NA=not assessed. For Bartel index, a higher score indicates greater degree of functional independence.

* Pre-stroke score.

† Higher score indicated higher burden.

There were no apparent differences between groups in any of the secondary outcomes (table 2), with the exception of GHQ-12 at 6 months and perceived burden in caregivers. The co-careldopa group reported better mood with a 1·3 point difference in the GHQ-12 at 6 months, compared with the placebo group, but not at 8 weeks or 12 months. Carers in the co-careldopa group reported less burden at 8 weeks (CBS mean difference −4·55 points [95% CI −0·14 to −8·96]; p=0·043), 6 months (–4·99 [–0·17 to −9·81]; p=0·042), and 12 months (–7·17 [–1·70 to −12·64]; p=0·011). Responses from carers were obtained in under half of study participants and numbers varied at each timepoint (table 2). During the 12-month follow up, the proportion of participants reporting no psychological distress seemed to improve from around a third to almost half of patients in both placebo and co-careldopa treated groups. An increase in the proportion of participants who were able to walk was observed at 6 months in both groups. Little further change was observed at 12 months in either of the groups. As expected, NEADL scores at 8 weeks after randomisation were lower than the prestroke scores in both groups (patients were asked their prestroke status). As for physical function, improvement in NEADL scores was seen at 6 months. ABILHAND scores showed a similar pattern of improvement. Good functional independence (mRS score of 0–2) at 8 weeks was achieved in less than a fifth of participants in both groups. Musculoskeletal pain was reported by two thirds of participants at 8 weeks in both groups (appendix).

Post-hoc subgroup analyses provided some evidence that participants with cerebral infarction might be less likely to recover walking ability (206 [41%] of 508 patients able to walk independently at 8 weeks) than those with intracerebral haemorrhage (46 [54%] of 85 patients; table 3). There was some evidence that ability to walk independently at 8 weeks might be associated with higher baseline scores for RMI, prestroke NEADL, and Barthel Index. There was also weak evidence that walking at 8 weeks could be inversely associated with age and time from stroke to randomisation. No evidence was found for an association between sex and independent walking at 8 weeks (169 [46%] of 364 men *vs* 83 [36%] of 229 women could walk independently).

**Table 3:** Variables included in the primary analysis and estimates from stepwise multilevel logistic regression analysis

		**Able to walk independently at 8 weeks (n=252)**	**Total (n=593)**	**Estimated odds ratio (95% CI)**	**p value**
Sex					
	Men	169 (46%)	364 (100%)	0·90 (0·60–1·35)	0·612
	Women	83 (36%)	229 (100%)	..	..
Mean age, years		65·5 (14·1)	68·5 (13·2)	0·98 (0·97–0·99)	0·010
Type of stroke					
	Infarction	206 (82%)	508 (86%)	0·38 (0·22–0·67)	0·001
	Primary haemorrhage	46 (18%)	85 (14%)	..	..
Mean RMI score at baseline‡		3·1 (1·8)	2·3 (1·8)	1·52 (1·31–1·76)	<0·0001
Mean Barthel Index at baseline		9·3 (3·4)*	7·7 (3·7)†	1·11 (1·03–1·19)	0·004
Pre-stroke NEADL score					
	Mean‡	60·7 (10·0)‡	58·8 (11·7)†	1·03 (1·01–1·05)	0·011
	Median	66 (0–66)	63 (0–66)	..	..
Days between stroke and randomisation					
	Mean	15·0 (9·6)	17·7 (10·1)	0·95 (0·93–0·97)	<0·0001
	Median	12 (3–59)	15 (3–59)	..	..

Data are n (%), mean (SD), median (range). RMI=Rivermead Mobility Index. NEADL=Nottingham Extended Activities Daily Living.

* Data missing in five patients.

† Data missing from 13 patients.

‡ Data missing in seven patients. Data missing at baseline.

132 serious adverse events were reported from 107 (18%; table 4) participants: 74 serious adverse events in 57 (19%) participants in the co-careldopa group and 58 in 50 (18%) participants in the placebo group. Most of the serious adverse events reported in both groups were not suspected to be related to the trial medication (2 [3%] in the co-careldopa group *vs* 1 [2%] in the placebo group were suspected to be related to treatment; appendix). No suspected unexpected serious adverse reactions were reported. Slightly more deaths occurred in the co-careldopa group the placebo group during the study (22 [7%] of 308 patients *vs* 17 [6%] of 285 patients; appendix). More participants in the co-careldopa group died within 8 weeks (6 [2%] *vs* 1 [0%]) but none of the deaths were considered likely to be related to study treatment. More participants in the co-careldopa group vomited during therapy after study drug administration (19 [6%] *vs* 9 [3%]). The primary economic analysis used multiple imputation for missing data and adjustment for baseline EQ-5D differences. On average, patients in the co-careldopa group incurred higher costs and gained fewer QALYs than patients in the placebo group, indicating that co-careldopa is not cost-effective. Bootstrapping results indicate that, at a willingness to pay threshold of £20 000 per QALY, co-careldopa has only a 7% chance of being cost-effective (appendix).

**Table 4:** Serious adverse events by MedDRA System Organ Classification

	**Co-careldopa (n=74)**	**Placebo (n=58)**
Blood and lymphatic system disorders	1 (1%)	0 (0%)
Cardiac disorders	6 (8%)	2 (3%)
Gastrointestinal disorders	4 (5%)	3 (5%)
General disorders and administration site conditions	0 (0%)	1 (2%)
Hepatobiliary disorders	1 (1%)	2 (3%)
Infections and infestations	10 (14%)	6 (10%)
Injury, poisoning, and procedural complications	4 (5%)	3 (5%)
Metabolism and nutrition disorders	2 (3%)	1 (2%)
Musculoskeletal and connective tissue disorders	1 (1%)	0 (0%)
Neoplasms benign, malignant, and unspecified (including cysts and polyps)	2 (3%)	3 (5%)
Nervous system disorders	10 (14%)	11 (19%)
Psychiatric disorders	2 (3%)	0 (0%)
Renal and urinary disorders	2 (3%)	1 (2%)
Reproductive system and breast disorders	1 (1%)	3 (5%)
Respiratory, thoracic, and mediastinal disorders	8 (11%)	12 (21%)
Social circumstances	4 (5%)	0 (0%)
Vascular disorders	16 (22%)	10 (17%)

Data are n (%).

## Discussion

The DARS study found no evidence of improvement in walking ability when combining dopaminergic therapy with motor therapy after acute stroke. Although just over 10% patients were lost to follow-up, the findings are likely to be robust and generalisable to patients with limited mobility in the first few weeks after stroke. A greater proportion of patients achieved the primary outcome of independent walking at 8 weeks in the placebo group than was anticipated in comparison to a previous study (44% *vs* 26%).^12^ This result is most likely to be due to recruitment of participants at an earlier time after stroke onset in DARS at an average of 18 days compared with 43 days.^12^ A range of secondary outcomes in DARS showed no suggestion of benefit on arm function, disability, activities of daily living, or cognition. Co-careldopa had an acceptable safety profile. Vomiting, a known side-effect of levodopa, was uncommon but more frequent with co-careldopa than with placebo.

Levodopa was chosen from several other potential drug therapies, such as amphetamines and selective serotonin reuptake inhibitors (SSRIs), because levodopa has a better safety profile than amphetamines and was considered to have stronger basis for a purported mechanism of action on motor learning than SSRIs.^23^

Strengths of the trial are the double-blind, placebo-controlled design, large participant recruitment from multiple NHS stroke services, and good adherence with study treatment and therapy sessions, with over 80% of participants receiving at least 20 min of motor therapy in over 80% of therapy sessions. Although 60% more placebo patients received sufficient motor therapy, fewer than 10% of patients met the prespecified per-protocol analysis criteria mainly due to participants taking the trial medication outside the specified 45–60 min window before therapy. This criterion was, in retrospect, too strict because serum levodopa concentration would be elevated at the target concentration for a wider time window of 30–120 min. Future trials of combined timed drug and therapy might consider the use of less strict criteria for per-protocol analyses.

A limitation of the trial was the loss to follow up at 8 weeks. The primary ITT analysis made the assumption that those participants with missing outcome data did not achieve the primary outcome (ie, walking independently). Sensitivity analyses testing the robustness of this assumption did not alter the ITT analysis conclusion.

A possible explanation for the absence of a significant response to co-careldopa is the use of intermittent rather than sustained daily dosing, as was used in previous trials, including a positive small trial^12^ that used 3 weeks' continuous levodopa therapy. An intermittent dosing strategy was chosen with the intention of maximising brain dopamine concentrations during therapy and reducing the risk of adverse effects and patient withdrawal. Higher doses of co-careldopa might be beneficial for some patients but could potentially lead to more adverse effects. The dose used in the trial produces clinical benefits in Parkinson's disease.^24^ Future phase 2 trials of recovery-enhancing drugs might usefully compare intermittent versus daily dosing in terms of tolerability and clinical measures of recovery.

Another possible hypothesis for the absence of response to co-careldopa is that the intensity of therapy delivered in DARS was insufficient. However, DARS participants received the 16 h recommended from a systematic review of augmented therapy time.^25^

DARS enrolled more participants with stroke than all previous studies of dopaminergic therapy combined. The results are consistent with these smaller studies.^6^, ^7^, ^8^, ^9^, ^10^, ^11^, ^12^ A formal meta-analysis of trials of dopaminergic therapy in stroke recovery has not been done and, given the wide range of different outcome measures in populations of patients with stroke recruited at different times after stroke, might not be appropriate. Of the seven reported randomised trials, three showed no benefit on motor function,^7^, ^10^, ^11^ two showed improvements in walking speed^8^ or procedural motor learning,^9^ and one slightly larger study showed a slight improvement in disability.^6^ One trial^26^ showed that 100 mg of levodopa administered daily had a benefit on walking in 53 participants, with a significant increase in Rivermead Motor Assessment score of 2·3 points after 3 weeks compared with placebo. This trial involved more frequent daily physical therapy.

Future research of pharmacotherapy and stroke recovery should consider incorporation of potential proof of concept imaging biomarkers, such as fMRI (laterality index) and transcranial magnetic stimulation (motor evoked potentials) measures, into early phase trials.^27^ Future research is needed into the development of more sensitive clinical markers of motor recovery that would show proof-of-concept efficacy on neurological impairment in early phase trials before doing large pragmatic trials with activity and disability measures as the primary trial outcome.

DARS is the largest, multicentre, stroke rehabilitation trial to combine timed administration of a masked investigational medicinal product with therapy sessions. This approach required a high degree of coordination of drug administration with planned therapy and was successfully delivered with support from research teams from the National Institute for Health Research Stroke Research Network. DARS has shown that it is feasible to deliver multicentre trials of pharmacotherapy-enhanced rehabilitation in NHS stroke services but highlights the challenges involved in multicentre trials in delivering combined drug and motor therapy at an intensity recommended by expert guidelines. Although we found no evidence of dopaminergic therapy in conjunction with motor therapy improving walking after stroke, the learning and experience from DARS has lessons for the design and conduct of future rehabilitation multicentre trials investigating the effect of drugs to enhance recovery.
