# Supplementary material for: Safety and efficacy of co-careldopa as an add-on therapy to occupational and physical therapy in patients after stroke (DARS): a randomised, double-blind, placebo-controlled trial
Source: Lancet Neurol. 2019 Jun;18(6):530–8. doi: 10.1016/S1474-4422(19)30147-4 (PMC6527868; doi:10.1016/S1474-4422(19)30147-4)
Supplement: Supplementary appendix [file mmc1.pdf]

THE LANCET
Neurology

Supplementary appendix

This appendix formed part of the original submission and has been peer reviewed. We post it as supplied by the authors.

Supplement to: Ford GA, Bhakta BB, Cozens A, et al. Safety and efficacy of co-careldopa as an add-on therapy to occupational and physical therapy in patients after stroke (DARS): a randomised, double-blind, placebo-controlled, trial. *Lancet Neurol* 2019; **18:** 530–38.

Dopamine Augmented Rehabilitation in Stroke (DARS): a multi-centre double-blind, randomised controlled trial of co-careldopa compared with placebo, in addition to routine NHS occupational and physical therapy, delivered early after stroke on functional recovery

Supplementary material

Table of Contents

1. Search strategy
2. Summary of therapy data: table 1
3. Compliance details and estimates with 95% CI for the ability to walk independently at 8 weeks amongst compliers: table 2
4. Asessment of pain (MSK-SSP Manikin)
5. Number and proportion of participants reporting pain on MSK-SSP Manikin: table 3
6. Summary of serious adverse events (SAEs): table 4
7. Summary of participant deaths: table 5
8. Cost-effectiveness results: table 6
9. Participant sites and principal investigators

1. Search strategy

Search strategy for Ovid Medline (1946 – 2015 September 25)					
**1**	exp Stroke/	**28**	exp Dihydroxyphenylalanine/	**55**	recover$.mp.
**2**	exp Stroke, Lacunar/	**29**	$dopa$.mp.	**56**	(motor adj3 function$).mp.
**3**	exp Cerebrovascular Disorders/	**30**	sinemet.mp.	**57**	(motor adj3 perform$).mp.
**4**	exp Cerebral Infarction/	**31**	$careldopa.mp.	**58**	(motor adj3 skill$).mp.
**5**	exp Brain Infarction/	**32**	madopar.mp.	**59**	(upper adj3 function$).mp.
**6**	exp Cerebrovascular Circulation/	**33**	24 or 25 or 26 or 27 or 28 or 29 or 30 or 31 or 32	**60**	(arm adj3 function$).mp.
**7**	exp Arterial Occlusive Diseases/	**34**	exp Rehabilitation/	**61**	(upper adj3 move$).mp.
**8**	exp Cerebral Hemorrhage/	**35**	exp "Recovery of Function"/	**62**	(hand adj3 function$).mp.
**9**	exp Brain Ischemia/	**36**	exp Physical Therapy Modalities/	**63**	(hand adj3 move$).mp.
**10**	exp Hemiplegia/	**37**	exp Walking/	**64**	(hand adj3 dexter$).mp.
**11**	exp Paresis/	**38**	exp Mobility Limitation/	**65**	(upper adj3 limb$).mp.
**12**	stroke.mp.	**39**	exp "Activities of Daily Living"/	**66**	(upper adj3 extrem$).mp.
**13**	(cerebr*vascular adj3 accident).mp.	**40**	exp Arm/	**67**	(leg adj3 function$).mp.
**14**	(cerebrovascular adj3 accident).mp.	**41**	exp Upper Extremity/	**68**	(leg adj3 move$).mp.
**15**	(cerebral adj2 vascular adj2 accident).mp.	**42**	exp Hand/	**69**	(lower adj3 limb).mp.
**16**	CVA.mp.	**43**	exp Hand Strength/	**70**	(lower adj3 extrem$).mp.
**17**	hemipleg*.mp.	**44**	exp Leg/	**71**	physi$ therapy.mp.
**18**	hemipar*.mp.	**45**	exp Lower Extremity/	**72**	physiotherapy.mp.
**19**	cereb* isch?emi*.mp.	**46**	exp Muscle Strength/	**73**	occupat$ therapy.mp.
**20**	cerebral h?emorrhage.mp.	**47**	exp Locomotion/	**74**	walk$.mp
**21**	intracerebral h?emorrhage.mp.	**48**	exp Gait/	**75**	gait.mp.
**22**	parenchymal h?emorrhage.mp.	**49**	exp Gait Disorders, Neurologic/	**76**	ambulat$.mp.
**23**	1 or 2 or 3 or 4 or 5 or 6 or 7 or 8 or 9 or 10 or 11 or 12 or 13 or 14 or 15 or 16 or 17 or 18 or 19 or 20 or 21 or 22	**50**	exp Gait Apraxia/	**77**	mobil$.mp.
**24**	exp Dopamine/	**51**	exp Motor Skills/	**78**	transfer$.mp.
**25**	exp Dopamine Agents/	**52**	exp Treatment Outcome/	**79**	34 or 35 or 36 or 37 or 38 or 39 or 40 or 41 or 42 or 43 or 44 or 45 or 46 or 47 or 48 or 49 or 50 or 51 or 52 or 53 or 54 or 55 or 56 or 57 or 58 or 59 or 60 or 61 or 62 or 63 or 64 or 65 or 66 or 67 or 68 or 69 or 70 or 71 or 72 or 73 or 74 or 75 or 76 or 77 or 78
**26**	exp Dopamine Agonists/	**53**	exp Social Participation/	**80**	**23 and 33 and 79**
**27**	exp Levodopa/	**54**	rehabilitat$.mp.		

Search strategy for Embase (1996 – 2014 Week 42) and Embase Classic (1947 – 2015 September 25)					
**1**	exp cerebrovascular accident/	**32**	21 or 22 or 23 or 24 or 25 or 26 or 27 or 28 or 29 or 30 or 31	**63**	exp social participation/
**2**	exp cerebrovascular disease/	**33**	exp rehabilitation/	**64**	exp social adaptation/
**3**	exp brain infarction/	**34**	exp convalescence/	**65**	exp social interaction/
**4**	exp brain hemorrhage/	**35**	exp physiotherapy/	**66**	exp occupational therapy/
**5**	exp hemiplegia/	**36**	exp home physiotherapy/	**67**	exp physical disability/
**6**	exp hemiparesis/	**37**	exp walking/	**68**	exp outcome assessment/
**7**	exp lacunar stroke/	**38**	exp walking difficulty/	**69**	exp treatment outcome/
**8**	exp brain ischemia/	**39**	exp walking speed/	**70**	exp outcomes research/
**9**	exp paresis/	**40**	exp locomotion/	**71**	exp rating scale/
**10**	stroke.mp.	**41**	exp gait/	**72**	rehabilitat$.mp.
**11**	lacun$.mp.	**42**	exp gait disorder/	**73**	recover$.mp.
**12**	($vascular adj3 accident$).mp.	**43**	gait/ or exp neurologic gait disorder/	**74**	arm.mp.
**13**	cerebrovascular accident.mp.	**44**	exp gait apraxia/	**75**	leg.mp.
**14**	cerebral vascular accident.mp.	**45**	exp hemiplegic gait/	**76**	(upper adj3 limb).mp.
**15**	hemipleg$.mp.	**46**	gait/ or exp unsteady gait/	**77**	(upper adj3 extremit$).mp.
**16**	hemipar$.mp.	**47**	gait/ or exp spastic gait/	**78**	(lower adj3 limb$).mp.
**17**	isch?emi$.mp.	**48**	exp limited mobility/	**79**	(lower adj3 extremit$).mp.
**18**	CVA.mp.	**49**	exp patient mobility/	**80**	mobil$.mp.
**19**	h?emorrhag$.mp.	**50**	exp physical mobility/	**81**	walk$.mp.
**20**	1 or 2 or 3 or 4 or 5 or 6 or 7 or 8 or 9 or 10 or 11 or 12 or 13 or 14 or 15 or 16 or 17 or 18 or 19	**51**	exp daily life activity/	**82**	ambulat$.mp.
**21**	exp dopamine/	**52**	exp arm/	**83**	$therapy.mp.
**22**	exp dopamine receptor stimulating agent/	**53**	exp arm movement/	**84**	outcome.mp.
**23**	exp levodopa/	**54**	exp arm exercise/	**85**	(arm adj3 function$).mp.
**24**	exp carbidopa plus levodopa/	**55**	arm/ or exp arm weakness/	**86**	(hand adj3 function$).mp.
**25**	exp carbidopa plus entacapone plus levodopa/	**56**	exp hand/	87	(leg adj3 function$).mp.
**26**	exp benserazide plus levodopa/	**57**	exp hand grip/	88	(upper limb adj3 function$).mp.
**27**	exp DOPA/	**58**	exp hand movement/	89	(lower limb adj3 function$).mp.
**28**	$dopa$.mp.	**59**	exp hand function/	90	33 or 34 or 35 or 36 or 37 or 38 or 39 or 40 or 41 or 42 or 43 or 44 or 45 or 46 or 47 or 48 or 49 or 50 or 51 or 52 or 53 or 54 or 55 or 56 or 57 or 58 or 59 or 60 or 61 or 62 or 63 or 64 or 65 or 66 or 67 or 68 or 69 or 70 or 71 or 72 or 73 or 74 or 75 or 76 or 77 or 78 or 79 or 80 or 81 or 82 or 83 or 84 or 85 or 86 or 87 or 88 or 89
**29**	sinemet.mp.	**60**	exp hand strength/ or hand/	**91**	**20 and 32 and 90**
**30**	$careldopa.mp.	**61**	exp leg/		
**31**	madopar.mp.	**62**	exp leg movement/		

Search strategy for Ovid PSYCHINFO (1806 – 2015 September 25)					
**1**	exp Cerebrovascular Accidents/	**23**	madopar.mp.	**45**	exp Physical Strength/
**2**	exp Cerebral Ischemia/	**24**	levodopa.mp.	**46**	exp Exercise/
**3**	exp Cerebrovascular Disorders/	**25**	L-dopa.mp.	**47**	exp Treatment Outcomes/
**4**	exp Cerebral Hemorrhage/	**26**	16 or 17 or 18 or 19 or 20 or 21 or 22 or 23 or 24 or 25	**48**	exp Rating Scales/
**5**	exp Hemiplegia/	**27**	exp Rehabilitation/	**49**	exp Measurement/
**6**	exp Hemiparesis/	**28**	exp Motor Processes/	**50**	rehabilitat$.mp.
**7**	Stroke.mp.	**29**	exp Motor Performance/	**51**	recover$.mp.
**8**	lacun$.mp.	**30**	exp Physical Therapy/	**52**	arm.mp.
**9**	cerebrovascular accident$.mp.	**31**	exp Occupational Therapy/	**53**	leg.mp.
**10**	cerebral vascular accident$.mp.	**32**	exp "Activities of Daily Living"/	**54**	(upper adj3 limb).mp.
**11**	$vascular accident$.mp.	**33**	exp Walking/	**55**	(lower adj3 limb).mp.
**12**	CVA.mp.	**34**	exp Gait/	**56**	(lower adj3 extremit$).mp.
**13**	hemipleg$.mp.	**35**	exp Physical Mobility/	**57**	(upper adj3 extremit$).mp.
**14**	hemipare$.mp.	**36**	exp Disabilities/	**58**	hand.mp.
**15**	1 or 2 or 3 or 4 or 5 or 6 or 7 or 8 or 9 or 10 or 11 or 12 or 13 or 14	**37**	exp Social Interaction/	**59**	mobil$.mp.
**16**	exp Dopamine/	**38**	exp Participation/	**60**	walk$.mp.
**17**	exp Dopamine Agonists/	**39**	exp Learning/	**61**	ambulat$.mp.
**18**	exp Levodopa/	**40**	exp Motor Coordination/	**62**	$therapy.mp.
**19**	exp DOPA/	**41**	exp "Arm (Anatomy)"/	**63**	outcome.mp.
**20**	$dopa$.mp.	**42**	exp "Hand (Anatomy)"/	**64**	27 or 28 or 29 or 30 or 31 or 32 or 33 or 34 or 35 or 36 or 37 or 38 or 39 or 40 or 41 or 42 or 43 or 44 or 45 or 46 or 47 or 48 or 49 or 50 or 51 or 52 or 53 or 54 or 55 or 56 or 57 or 58 or 59 or 60 or 61 or 62 or 63
**21**	sinemet.mp.	**43**	exp Grasping/	**65**	**15 and 26 and 64**
**22**	$careldopa.mp.	**44**	exp "Leg (Anatomy)"/		

Summary of therapy data

Table 1 Summary of IMP doses, motor therapy sessions and time frame of IMP in relation to therapy sessions

	Co-careldopa (n=7319)	Placebo (n=7232)	Total (n=14551)
**Number of motor therapy sessions**			
Mean (SD)	23·2 (14·36)	24·8 (12·50)	24·0 (13·51)
**Number of IMP doses taken**			
Mean (SD)	20·6 (13·07)	22·4 (11·10)	21·5 (12·18)
**IMP taken (N sessions (%))**			
45-60 minutes before motor therapy	4030 (55·1%)	3976 (55·0%)	8006 (55·0%)
>1 hour before motor therapy	1079 (14·7%)	1074 (14·9%)	2153 (14·8%)
<45 minutes before motor therapy	805 (11·0%)	885 (12·2%)	1690 (11·6%)
not taken before motor therapy	1205 (16·5%)	1031 (14·3%)	2236 (15·4%)
after start of motor therapy	100 (1·4%)	154 (2·1%)	254 (1·7%)
unknown time before/after motor therapy	77 (1·1%)	73 (1·0%)	150 (1·0%)
missing study drug or therapy data	23 (0·3%)	39 (0·5%)	62 (0·4%)

Compliance

All patients received their treatment in accordance with their randomised allocation. Of the 593 patients with primary outcome data, 536 had 5 or more therapy sessions and were included in descriptive compliance statistics.

The primary analysis was by ITT; subsequently further analyses assessed the sensitivity of the conclusions of this analysis to non-compliance, using a staged definition of "compliance" (binary: yes, no). This definition was used for the purpose of complier-average causal effect (CACE) analyses. This was based on whether the drug was taken and the timing of this, the amount of motor therapy and the number of sessions:

1) Strict compliance – randomised drug taken, 45-60 minutes before therapy, involving 20 minutes or more of motor therapy, for 80% or more of the sessions.
2) Relaxed timing compliance – randomised drug taken 0-60 minutes or within 3 hours of a dose of drug (if patient had two therapy sessions directly one after the other) before therapy, involving 20 minutes or more motor therapy, for 80% or more of the sessions.
3) Relaxed timing and motor therapy compliance – randomised drug taken 0-60 minutes or within 3 hours of a dose of drug (if patient had two therapy sessions directly one after the other) before therapy for 80% or more of the motor sessions.
4) Drug intake compliance – 20 minutes or more of motor therapy for 80% or more of the sessions and missed 20% or less of drug within therapy sessions involving 20 minutes or more of motor therapy randomised drug taken at least 80% of the time.

If less than five therapy sessions had been arranged, then the definitions of compliance were set to missing. Treatment withdrawals were handled within the above definitions.

Table 2 Compliance and estimates with 95% CI for the ability to walk independently at 8 weeks amongst compliers

Stage of compliance<br><br>Complied (N, %)	Placebo (n=265)		Co-careldopa (n=271)		OR (95% CI)	p-value
	N (% out of n)	Walking independently at 8 weeks (N1, % out of N)	N (% out of n)	Walking independently at 8 weeks (N1, % out of N)		
Strict compliance	32 (12·1)	12 (37·5)	39 (14·4)	19 (48·7)	0·868 (0·282, 2·676)	0·806
Relaxed timing compliance	98 (37·0)	49 (50·0)	91 (33·6)	46 (50·6)	0·701 (0·354, 1·391)	0·310
Relaxed timing and therapy compliance	116 (43·8)	58 (50·0)	115 (42·4)	57 (49·8)	0·769 (0·412, 1·437)	0·411
Relaxed drug intake compliance	202 (76·2)	90 (44·6)	185 (68·3)	81 (43·8)	0·882 (0·540, 1·440)	0·615

Treatment effect amongst compliers was assessed by including an interaction between treatment group and compliance in the primary analysis multilevel model. The primary multilevel model adjusted for baseline RMI, age, gender, stroke type, baseline BI, baseline NEADL score, and days between stroke and randomisation, and included a random intercept for site.

Assessment of pain (MSK-SSP Manikin)
Musculoskeletal Symptoms/Signs and Pain manikin: The MSK-SSP manikin uses tick boxes to allow for self-reporting on areas of pain. There are 20 locations or tick boxes on the manikin. There is no standard approach available for scoring this measure. For this trial, a number of dichotomous variables was derived based on a combination of locations on the pain manikin:

1. Patients with or without pain in upper limbs; including thumb, hand/fingers, wrist, elbow and shoulder. Pain indicated in any of these locations will be categorised as pain in the upper limb.

2. Patients with or without pain in lower limbs; including hip, knee, ankle and feet/toes. Pain indicated in any of these locations will be categorised as pain in the lower limb.

3. Central post-stroke pain will be categorised as such, if there is pain reported in all loci in the entire limb or hemi-section of the body (i.e. all the loci on a particular limb or half-body within the manikin are ticked).

4. Pain in the thumb, hand/ fingers, and wrists joints.

5. Pain in the back and neck will be categorised as spinal pain.

The decision to combine pain locations in this way was based on how MSK-SSP has previously been used and on estimates of pain prevalence in a similar stroke population

Table 3 Number and proportion of participants reporting pain on MSK-SSP Manikin

Assessment (N (%))	Baseline		8 weeks		6 months		12 months	
	Co-careldopa	Placebo	Co-careldopa	Placebo	Co-careldopa	Placebo	Co-careldopa	Placebo
MSK-SSP Manikin								
Joint, neck or back pain[‡]	129 (41·9)	107 (37·5)	206 (66·9)	191 (67·0)	188 (61·0)	187 (65·6)	152 (49·4)	146 (51·2)
Pain in upper limbs[‡]	60 (19·5)[¶]	54 (18·9)[¶]	165 (53·6)	159 (55·8)	148 (48·1)	138 (48·4)	113 (36·7%)	108 (37·9)
Pain in lower limbs[‡]	82 (26·6)[¶]	72 (25·3)[¶]	127 (41·2)	113 (39·6)	126 (40·9)	130 (45·6)	117 (38·0%)	100 (35·1)
Right central post-stroke pain[‡]	6 (1·9)[¶]	2 (0·7)[¶]	14 (4·5)	11 (3·9)	10 (3·2)	13 (4·6)	11 (3·6%)	10 (3·5)
Left central post-stroke pain[‡]	3 (1·0)[¶]	1 (0·4)[¶]	19 (6·2)	22 (7·7)	19 (6·2)	17 (6·0)	19 (6·2%)	14 (4·9)
Central post-stroke pain[‡]	7 (2·3)[¶]	3 (1·1)[¶]	31 (10·1)	32 (11·2)	26 (8·4)	30 (10·5)	29 (9·4%)	22 (7·7)
Right thumb/hand/fingers/wrist joint pain[‡]	5 (1·6)[¶]	3 (1·1)[¶]	16 (5·2)	20 (7·0)	8 (2·6)	13 (4·6)	17 (5·5%)	6 (2·1)
Left thumb/hand/fingers/wrist joint pain[‡]	6 (1·9)[¶]	3 (1·1)[¶]	37 (12·0)	33 (11·6)	22 (7·1)	18 (6·3)	17 (5·5%)	13 (4·6)
Any thumb/hand/fingers/wrist joint pain[‡]	8 (2·6)[¶]	5 (1·8)[¶]	52 (16·9)	48 (16·8)	29 (9·4)	31 (10·9)	32 (10·4%)	18 (6·3)
Spinal pain[‡]	10 (3·2)[¶]	5 (1·8)[¶]	3 (1·0)	3 (8·8)	2 (0·6)	5 (1·8)	6 (1·9%)	8 (2·8)

[‡]N=yes (%);[¶†]Pre-stroke score

Safety

Serious adverse events (SAEs)

Table 4 Summary of serious adverse events (SAEs)

	Co-careldopa (n=308)	Placebo (n=285)	Total (n=593)
**Number of SAEs reported**	74	58	132
**Number of participants with one or more SAEs N(%)**	57 (18·5%)	50 (17·5%)	107 (18·0%)
**Number of SAEs per participant**			
Mean (SD)	1·3 (0·68)	1·2 (0·42)	1·2 (0·58)
Median (Range)	1·0 (1·0, 4·0)	1·0 (1·0, 3·0)	1·0 (1·0, 4·0)
**Suspected to be related to trial medication (out of SAEs reported) N(%)**	2 (2·7%)	1 (1·7%)	3 (2·3%)

Deaths

Table 5 Summary of participant deaths

	Co-careldopa (n=308)	Placebo (n=285)	Total (n=593)
**Number of deaths**	22 (7·1%)	17 (6·0%)	39 (6·6%)
**Time death occurred**			
Up to 8 weeks	6 (1·9%)	1 (0·4%)	7 (1·2%)
From 8 weeks to 6 months	7 (2·3%)	7 (2·5%)	14 (2·4%)
From 6 months to 12 months	9 (2·9%)	9 (3·2%)	18 (3·0%)
**Place of death**			
Home	2 (0·6%)	4 (1·4%)	6 (1·0%)
Hospital	14 (4·5%)	12 (4·2%)	26 (4·4%)
Institutional care	5 (1·6%)	2 (0·7%)	7 (1·2%)
Unknown	3 (1·0%)	2 (0·7%)	5 (0·8%)

Table 6 Cost-effectiveness results (wider perspective for costs, i.e. include healthcare provider costs and patient costs)

	Costs (£)	QALY*	
	Mean (SD)	*Mean (SD)*	
Placebo	15,266·73 (16987·16)	0·421 (0·030)	
Co-careldopa	17,484·66 (18579·82)	0·407 (0·281)	
	**Incremental cost**	**Incremental QALY**	**ICER (£/QALY)**
Co-careldopa *vs* placebo	£2217·93	-0·022	Co-careldopa dominated

*QALYs are adjusted for EQ-5D differences at baseline

Participant sites and principal investigators

Aintree University Hospital NHS Foundation Trust (Dr Claire Cullen)

Northern Health & Social Care Trust (Dr Jamil Vhidassr)

Heart of England NHS Foundation Trust (Dr David Sandler)

Blackpool Teaching Hospitals NHS Foundation Trust (Dr James McIlmoyle)

Bradford Teaching Hospitals NHS Foundation Trust (Dr Chris Patterson)

University Health Board, NHS Wales (Dr Phil Jones)

Chelsea & Westminster Hospital NHS Foundation Trust (Dr Michael Pelly)

Croydon Health Services NHS Trust (Dr Enas Lawrence)

East Sussex Healthcare NHS Trust (Dr Mudali Conrad)

Pennine Acute Hospitals NHS Trust (Dr Narayanamoorti Saravanan)

NHS Grampian (Dr Alastair Cozens)

NHS Greater Glasgow & Clyde (Dr Christine McAlpine)

Gloucestershire Hospitals NHS Foundation Trust (Dr Katharina Nehrig)

Harrogate and District NHS Foundation Trust (Dr Sean Brotheridge)

Staffordshire & Stoke on Trent Partnership NHS Trust (Prof Tony Ward)

Leeds Teaching Hospitals NHS Trust (Dr Peter Wanklyn)

Mid Cheshire Hospitals NHS Foundation Trust (Dr Maqsud Salehin)

Mid Yorkshire NHS Trust (Dr Prabal K Datta)

The Royal Wolverhampton NHS Trust (Dr Kenneth Fotherby)

NHS Tayside (Dr Ronald S MacWalter)

University Hospital of North Staffordshire NHS Trust (Prof Christine Roffe)

Northampton General Hospital NHS Trust (Dr Melanie Blake)

Portsmouth Hospitals NHS Trust (Dr Ugnius Sukys)

Burton Hospitals NHS Foundation Trust (Dr Partha Das)

Rotherham Doncaster & South Humber NHS Foundation Trust (Dr James Okwera)

East Lancashire Hospitals NHS Trust (Dr Mahiswar Goorah)

Royal Liverpool & Broadgreen University Hospitals NHS Trust (Dr Shnakar Loharuka)

Barts Health NHS Trust (Dr Rob Simister)

Belfast Health & Social Care Trust (Dr Ken Fullerton)

The Newcastle Upon Tyne Hospitals NHS Foundation Trust (Dr Anand Dixit)

North Lincolnshire & Goole NHS Foundation Trust (Dr Amit Banerjee)

Heart of England NHS Foundation Trust (Dr Uzma Khan)

University Hospital Southampton NHS Foundation Trust (Dr Nic Weir)

St George's Healthcare NHS Trust (Dr Geoffrey Cloud)

Epsom & St Helier NHS Trust (Dr Paul Omahony)

Guy's & St Thomas' NHS Foundation Trust (Dr Ajah Bhalla)

South Eastern Health & Social Care Trust (Dr Kevin Dynan)

Walsall Healthcare NHS Trust (Dr Elliot Epstein)

Weston Area Health NHS Trust (Dr Michael Haley)

St Helens & Knowesley Hospitals NHS Trust (Dr Tom Smith)

York Teaching Hospital NHS Foundation Trust (Dr Paul Willcoxson)

NHS Fife (Dr Vera Cvoro)

Northumbria NHS Trust (Dr Chris Price)

Southern Health and Social Care Trust (Dr Patricia McCaffrey)

County Durham and Darlington NHS Trust (Dr Ali Mehrzad)

Imperial Trust, London (Dr Paul Bentley).
